# Systematic Review on Surgical and Nonsurgical Treatment of Type II Odontoid Fractures in the Elderly

**DOI:** 10.1155/2014/231948

**Published:** 2014-02-10

**Authors:** Yohan Robinson, Anna-Lena Robinson, Claes Olerud

**Affiliations:** Department of Surgical Sciences, Uppsala University Hospital, 751 85 Uppsala, Sweden

## Abstract

Odontoid fractures type II according to Anderson and d'Alonzo are not uncommon in the elderly patients. Still, due to the paucity of evidence the published treatment guidelines are far from equivocal. This systematic review focuses on the published results of type II odontoid fracture treatment in the elderly with regard to survival, nonunion, and complications. After a systematic literature research 38 publications were included. A cumulative analysis of 1284 published cases found greater survival if elderly patients with odontoid fractures type II received surgical treatment (RR = 0.64). With regard to nonunion in 669 published cases primary posterior fusion had the best fusion results. The systematic literature review came to the following conclusions. (1) Surgical stabilisation of odontoid fractures type II improves survival in patients between 65 and 85 years of age compared to nonsurgical treatment. (2) Posterior atlantoaxial fusion for odontoid fractures type II in the elderly has the greatest bony union rate. (3) Odontoid nonunion is not associated with worse clinical or functional results in the elderly. (4) The complication rate of nonsurgical treatment is similar to the complication rate of surgical treatment of odontoid fractures type II in the elderly.

## 1. Background

Odontoid fractures type II according to Anderson and d'Alonzo [1] are typically related either to major trauma [2] or osteoporotic bone quality [3]. Due to the characteristics of the two injury mechanisms the incidence has a double peak distribution with fractures related to major trauma occurring in the younger patients, while osteoporotic fractures occur commonly in the elderly [4–6]. Odontoid fractures of the elderly related to osteoporosis often have intact C1-C2 joint capsules, anterior longitudinal ligament, and accessory ligaments, which stabilise the osteoporotic type II odontoid fracture produced in a low-energy mechanism [7]. Therefore type II odontoid fractures in the young and in the elderly are two different injuries, and different treatment protocols may be necessary.

While noncomminute odontoid fractures type II related to cervical high energy trauma are in many cases treated straightforward by anterior lag-screw osteosynthesis and a cervical collar for 6 weeks [8], the choice of treatment of osteoporotic odontoid fractures in the elderly has no consensus and is rather dependent on the region of the hospital the patient is admitted to than on scientific evidence [9]. In the USA a trend towards increased surgical treatment was identified [10], while in Sweden surgeons tend to prefer nonsurgical treatment especially in the elderly (unpublished data).

In 2013 four new retrospective cohort studies and one prospective study with altogether more than 400 cases were published on the treatment of odontoid fractures in the elderly [11–15]. It is therefore appropriate to revisit the published evidence on odontoid fracture treatment in the elderly.

This systematic review aims to represent the current evidence on the treatment of type II odontoid fractures in the elderly with regard to the following clinically important questions.

1. Does surgical stabilisation of odontoid fractures type II in elderly improve patient survival?
2. Which stabilisation method has the greatest success with regard to type II odontoid fracture healing?
3. Is odontoid nonunion associated with worse clinical results in the elderly?
4. What is the complication rate of nonsurgical treatment compared to surgical treatment of odontoid fractures type II in the elderly?

## 2. Methods

A comprehensive systematic literature review was performed using MeSH keywords with the search text “odontoid AND fracture AND elderly” in NLM PubMed MEDLINE, Ovid Medline, and ISI Web of Knowledge with the following inclusion and exclusion criteria.

### 2.1. Inclusion Criteria

Then following inclusion criteria were applied:

- acute odontoid fracture type II treatment (or separate data identifiable in manuscript),
- surgical and/or nonsurgical treatment,
- study of >10 cases (nonelderly included),
- elderly group (>60 or older) analysed separately or identifiable in manuscript,
- published between 1985 and October 2013,
- radiographic and/or clinical results and/or survival data.

### 2.2. Exclusion Criteria

The following exclusion criteria were applied:

- no separate analysis of type II odontoid fractures possible,
- elderly group not analysed separately or not identifiable in the paper,
- case series <10 cases (nonelderly included),
- review articles or experimental studies.

All included publications were summarised in Table 1 depicting age definition of the elderly and sample size of treatment groups. The quality of the available evidence was graded according to the GRADE criteria [16].

### 2.3. Statistics

The Statistical Package for Social Sciences (SPSS) version 21.0 by IBM (USA) was applied to perform the statistical analysis.

## 3. Results

### 3.1. Inclusion

**Figure 1:**
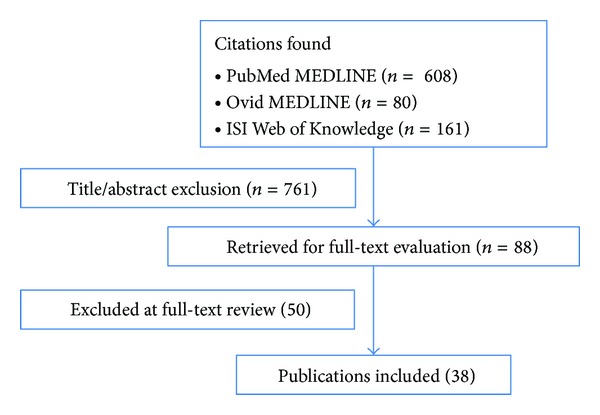
Flow chart depicting results from literature research in PubMed MEDLINE, Ovid MEDLINE, and ISI Web of Knowledge (MeSH terms: “odontoid,” “fracture,” and “elderly”).

The search of PubMed MEDLINE resulted in 608 citations, the search in Ovid MEDLINE in 80 citations, and the search in ISI Web of Knowledge in 161 citations related to the search keywords. 88 were left after abstract text review. After excluding 50 articles in full-text review, 38 publications met the inclusion criteria (Figure 1). All included studies except one prospective follow-up study [47–49] were retrospective cohort studies (Table 1).

### 3.2. Survival Analysis

In the full text of the included publications mortality data was identified and added to a cumulative database. In 29 articles mortality data from 1284 cases was available [3, 5, 6, 11–15, 18–21, 24–27, 31–36, 41–44, 46, 50]. If a patient characteristics chart was available, survival, follow-up, patient age, and treatment allocated were added directly to the database for each patient. If patient-specific data was missing, mean values for survival and patient age from the publication were entered into the database as a repeated entry—as many times as the number of included patients.

**Figure 2:**
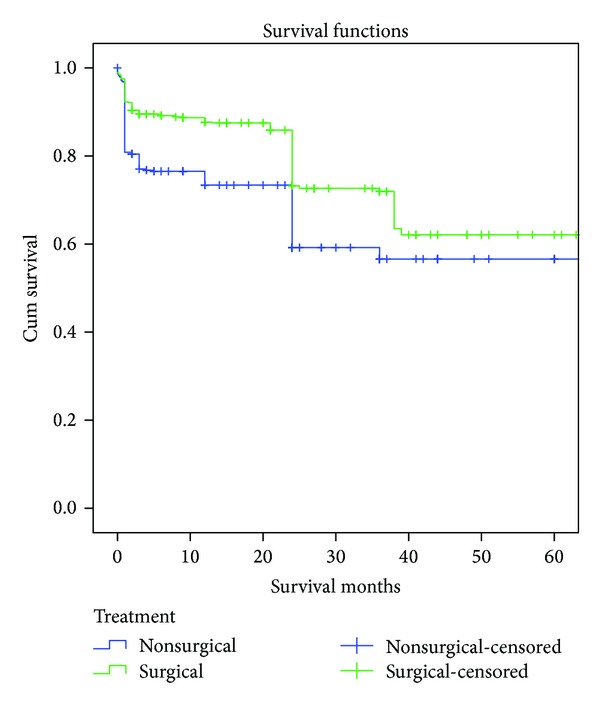
Kaplan-Meier survival functions for included cases with surgically and nonsurgically treated odontoid fractures type II in the elderly (*n* = 1284).

The Kaplan-Meier analysis revealed a mean survival of 83 months (95% CI: 78–88) (Table 2). The survival curve is plotted in Figure 2. Since several included studies, where only survival for a certain observational period of time was available, had an endpoint of 24 months, a distinct step is noticeable in the nonsurgical and the surgical survival curves at 24 months survival, indicating the artificial nature of this database. The survival curve reveals a high mortality rate in the nonsurgically treated group during the first months, which is maintained for up to three years. In general surgical treatment was associated with improved survival compared to nonsurgical treatment (Cox regression: surgical treatment RR 0.64, *P* < 0.001; patient age RR = 1.11, *P* < 0.001).

### 3.3. Fusion Rate

Data on fusion rate of nonsurgical and surgical treatment of 669 type two odontoid fractures was available in 29 included articles [3, 5, 6, 13–15, 17, 19–29, 31–33, 35–38, 40–43, 45, 46, 48, 49]. For each included study the number of patients allocated for each treatment and the number of nonunions that occurred until final follow-up were entered into a database. Summing up the included cases and nonunions a collective nonunion rate could be calculated for each treatment modality (Table 3). Nonsurgical treatment with collar or halo had high nonunion rates with 39% and 41%, respectively. Posterior fusion seems to be superior compared to anterior screw osteosynthesis with regard to bony union (11% versus 27% nonunion).

### 3.4. Clinical Results of Nonunion

Only 2 of the included studies focused on the clinical results of odontoid nonunion in the elderly [14, 48]. The subgroup analysis of the clinical and functional results of nonsurgically treated odontoid fractures in the elderly by the AOSpine North America Geriatric Odontoid Fracture (GOF) Study (*n* = 50) identified a nonunion in 11 patients [48]. No significant differences with regard to nonunion or union were found for Neck Disability Index (NDI) or SF-36 (including subscore analysis) both at baseline and 1-year follow-up. Molinari et al. [14] presented the functional outcome of 26 elderly patients with odontoid fractures treated with posterior fusion. No significant difference in NDI was found with regard to radiographical fusion or nonunion.

### 3.5. Complication Rate

For a proper estimation of the treatment complication rate a prospective study design is mandatory. Only the AOSpine NA GOF trial fulfils this requirement [49]. There was a nonsignificant trend towards a greater proportion of subjects with any complication in the nonsurgical group (36% versus 30%; *P* = 0.48). Surgically treated patients had a greater proportion of dysphagia compared to nonsurgically treated patients (11% versus 5%; *P* = n.s.). Since no subgroup analysis of the surgical treatment allocation was performed in that study, the presumptive association of anterior screw fixation and postoperative dysphagia remains hypothetical.

## 4. Discussion

### 4.1. Quality of Included Studies

Despite the large number of publications with regard to treatment of type II odontoid fractures in the elderly, most included studies only met the requirements of a “low” or “very low” quality of evidence [16]. Only the AOSpine NA GOF study could provide “moderate” quality evidence [12].

A selection bias with more nonsurgical patients lost to follow-up must be assumed, since in contrast to nonsurgical patients follow-up occurs naturally in the surgical group for implant stability control. Another possible selection bias is the (anaesthesiologist-driven) tendency to prefer nonsurgical treatment in the more morbid patient, while in healthier patients the decision for surgical treatment comes easier, which biases the morbidity and mortality data. Interestingly the prospective part of the North American AOSpine GOF study did not find any significant baseline differences between nonsurgically and surgically treated patients [49].

Furthermore a reporting bias in favour of new developments in surgical techniques and an underreporting of results of nonsurgical treatment cannot be excluded. Still, the availability of a large number of cases in the literature allows a cumulative estimation of mortality and bony union rates.

### 4.2. Does Surgical Stabilisation of Odontoid Fractures in the Elderly Improve Patient Survival?

Odontoid fractures in the elderly are obviously different from odontoid fractures in the younger patients. Due to the lesser impact causing the odontoid fracture in elderly patients, associated ligamentous injuries, which may lead to further dislocation, are relatively rare. Thus one could reason that surgical stabilisation of odontoid fractures is overtreatment in elderly patients. Since cervical immobilisation in a halo-vest or a collar is associated with an increased rate of pneumonia, pulmonary embolism and deep venous thrombosis, others fear instead the increased morbidity and mortality associated with nonsurgical treatment [51]. Both attitudes towards odontoid fracture treatment in the elderly are well represented in the medical community.

During the last decades increasing evidence has been collected implying a higher mortality rate in nonsurgically treated patients. The cumulative survival data presented in this review including 1284 elderly patients with odontoid fractures type II revealed a 20 months longer mean survival, if patients were treated surgically. Unfortunately this data could only be adjusted for age as possible confounder but not for gender, associated injuries, neurological status, and comorbidity.

The largest retrospective cohort published so far on odontoid fractures in the elderly is the AOSpine North America Geriatric Odontoid Fracture Mortality Study with 322 included patients [12]. After adjusting for the effects of patient age, sex, and comorbidities, surgically treated patients (*n* = 165) had a significantly better 30-day survival rate compared to nonsurgically treated patients (*n* = 157) (RR = 3.0; 95% CI: 1.51–5.94; *P* = 0.0017), and this effect prevailed until the final follow-up (RR = 1.35; 95% CI: 0.97–1.89; *P* = 0.079).

Even Schoenfeld et al. [44] found in their retrospective cohort of 156 patients increased 3-month and 1-year mortality in the nonsurgical group without reaching statistical significance (both *P* = 0.06). The authors then subdivided their cohort in three age groups. Interestingly improved survival with surgical treatment was most impressive in the group between 65 and 74 years of age (RR = 0.4; 95% CI: 0.1–1.5) and lesser in the group between 75 and 84 years of age (RR = 0.8; 95% CI: 0.3–2.3). Patients above the age of 85, instead, have possibly a greater mortality if treated surgically (RR = 1.9; 95% CI: 0.6–6.1).

Other factors than treatment modality seem to play a greater role for patient survival. Patel et al. [50] found in their investigation of 20 elderly patients with type II odontoid fractures associated with neurological deficit a higher mortality (RR = 4.7; 95% CI: 1.4–16.6) than in neurologically intact patients (*n* = 188); in patients with complete tetraparesis (*n* = 11) the risk was even higher (RR = 9.3; 95% CI: 1.2–73.0).

With regard to survival surgical treatment seems to be favourable over nonsurgical treatment in patients between 65 and 85 years of age.

 

*Quality of Evidence: moderate.*

There is conflicting evidence that surgical treatment for patients over the age of 85 is associated with greater mortality than nonsurgical treatment.

 
*Quality of Evidence: very low.*

### 4.3. Which Stabilisation Method Has the Greatest Success with Regard to Fracture Healing? 

Most studies published in the 1980s and 1990s are focusing on radiographical results of odontoid fracture treatment. On one hand this is caused by the greater availability of radiographical images in the hospital archives compared to clinical and functional scores, which were not widely employed, yet. On the other hand there is a strong (orthopaedic) belief that radiographical healing is associated with good clinical results [40]. The bony union rate of different treatment modalities of odontoid fractures is therefore in contrast to clinical and functional results well documented. Radiographical follow-up results on 669 patients with odontoid fractures could be extracted from the included articles and the results were clearly in favour of the surgical methods (Table 3). Obviously a primary posterior fusion leads to the greatest healing rate and thus the greatest stability of odontoid fractures in the elderly. Unfortunately only few studies used computed tomography to evaluate the nonunion rate, and most studies rely on stability in dynamic flexion-extension radiographs. Therefore an underreporting of the nonunion rate must be assumed, suggesting a significant source of bias.

With regard to bony union of odontoid fractures posterior fusion is superior to anterior screw osteosynthesis, which is superior to collar treatment, which is superior to halo-vest treatment.

 
*Quality of Evidence: low.*

### 4.4. Is Odontoid Nonunion Associated with Worse Clinical Results in the Elderly? 

After reviewing the published bony union rates of different treatment modalities, now the clinical relevance of bony union of the fractures odontoid in the elderly will be questioned. Only little has been published in this regard, and until recently only anecdotal case reports were available on the clinical results of radiographic odontoid nonunion. With regard to cervical fusion one retrospective cohort study on anterior cervical nonunion reported that 33% of patients with anterior nonunion were asymptomatic [52]. Still the good results of revision surgery in cervical nonunion suggest at least some biomechanical components in persistent postoperative pain of anterior cervical fusion [53].

One feared complication of odontoid nonunion is the development of myelopathy due to odontoid dislocation. Crockard et al. [54] presented a series of 16 cases with delayed presentation of myelopathy due to odontoid nonunion and central spinal stenosis caused by odontoid fragment dislocation. Interestingly they found myelopathy often to occur several years after the initial trauma. Still only 2 patients in their case series were older than 60 years, implying that myelopathy may not be as common in the elderly as in the young patient with odontoid fracture nonunion. In the case series by Paradis and Janes [55] all 29 patients with odontoid nonunion received surgical treatment, of which none was older than 70 years. Hart et al. [56] followed 5 elderly patients with odontoid nonunion without myelopathy (mean follow-up 4.6 years). None of these patients developed myelopathy or required surgical treatment.

Recently two studies were published investigating the effect of nonunion on the clinical and functional outcome of patients. The nonsurgical subgroup analysis of the prospective North American AOSpine GOF study by Smith et al. [48] found very similar clinical and functional results for patients achieving bony union (*n* = 39) and those with nonunion (*n* = 11). Even the retrospective study by Molinari et al. [14] found no statistically significant difference in functional outcome between the elderly patients with odontoid nonunion (*n* = 26) and the patients who achieved odontoid fracture healing (*n* = 7) after atlantoaxial posterior fixation (*P* = 0.5).

Obviously odontoid (fibrous) nonunion in the elderly is not associated with worse clinical outcome. Still, the anecdotal reports of delayed myelopathy in the elderly with odontoid nonunion suggest a minor risk for further odontoid dislocation, which must be assessed individually.

 
*Quality of Evidence: low.*

### 4.5. Is Surgical Treatment Associated with More Complications Compared to Nonsurgical Treatment of Odontoid Fractures Type II in the Elderly?

Despite the high relevance of this question, the current evidence does not favour any treatment modality for odontoid fractures type II in the elderly. The only prospective study in this regard found a trend towards more complications in the nonsurgical group, which failed to reach significance [49].

Of course there are method-related complications, which are well documented in retrospective case series. Cervical immobilisation in a collar is associated with 10% pressure ulcers [36]. Halo-vest immobilisation may be complicated in 4% by pin-site infection [26] and in 6% by pressure ulcers [36]. Anterior screw fixation was found to be associated with dysphagia in 17% to 35% [19, 42] and pneumonia in 14%–19% [3, 42], while posterior fusion was associated with postoperative infections in 33% [14] and pneumonia in 17% [35].

With regard to the increased mortality of patients receiving nonsurgical treatment, the complications occurring during and after nonsurgical treatment must obviously be more fatal, than those occurring after surgical treatment. The only published prospective trial failed to support this hypothesis, which may be due to a statistical type II error.

None of the available treatment modalities for odontoid fractures in the elderly was superior with regard to complications.

 
*Quality of Evidence: low.*

## 5. Conclusions

The systematic review of the published evidence on odontoid fracture treatment in the elderly allows following conclusions.

- Surgical stabilisation of odontoid fractures type II improves survival in patients between 65 and 85 years of age compared to nonsurgical treatment (quality of evidence: moderate).
- Primary posterior fusion for odontoid fractures type II in the elderly has the highest bony union rate (quality of evidence: low).
- Odontoid nonunion is not associated with worse clinical or functional results in the elderly (quality of evidence: low).
- The complication rate of nonsurgical treatment is similar to the complication rate of surgical treatment of odontoid fractures type II in the elderly (quality of evidence: low).

## Figures and Tables

**Table 1:** Articles included in the systematic review.

Author	Elderly	*N* all	*N* collar	*N* cast	*N* halo	*N* anterior	*N* posterior	Minimum follow-up
Pepin et al. (1985) [5]	>60	6		4	2			5 years
Dunn and Seljeskog (1986) [17]	>65	9			9			6 mo
Lind et al. (1987) [18]	>65	2			2			3 mo
Montesano et al. (1991) [19]	>65	6				6		5 mo
Jeanneret and Magerl (1992) [20]	>65	2					2	12 mo
Hanigan et al. (1993) [21]	>80	16	9		2		5	5 mo
Ryan and Taylor (1993) [22]	>60	14	4	9			1	6 mo
Polin et al. (1996) [23]	>60	16	16					3 mo
Berlemann and Schwarzenbach (1997) [24]	>65	19				19		4 mo
Seybold and Bayley (1998) [25]	>60	19	3		9		7	2 mo
Stoney et al. (1998) [26]	>70	11			11			12 mo
Müller et al. (1999) [6]	>70	22	14		3	5		18 mo
Kuntz 4th. et al. (2000) [27]	>65	20	3	3	8		6	3 mo
Andersson et al. (2000) [28]	>65	24	6		1	10	7	24 mo
Ziai and Hurlbert (2000) [29]	>65	43	31			8	4	3 mo
Bórm et al. (2003) [30]	>70	15					15	11 mo
Cornefjord et al. (2003) [31]	>65	14					14	3 mo
Frangen et al. (2007) [32]	>63	27					27	3 mo
Platzer et al. (2007) [33]	>65	41				41		12 mo
Smith et al. (2008) [34]	>80	72	24		16	10	22	1 mo
Kaminski et al. (2008) [35]	>70	36					36	24 mo
Koech et al. (2008) [36]	>65	42	10		32			9 mo
Štulik et al. (2008) [37]	>65	20				11	9	18 mo
Omeis et al. (2009) [38]	>70	29				16	13	3 mo
Fagin et al. (2010) [39]	>60	108	64		4	26	14	1 mo
Butler et al. (2010) [40]	>65	14	14					30 mo
Chaudhary et al. (2010) [41]	>70	20	9			11		3 mo
Dailey et al. (2010) [42]	>70	54				54		3 mo
Hou et al. (2011) [43]	>65	43				43		18 mo
Osti et al. (2011) [3]	>65	33				33		24 mo
Schoenfeld et al. (2011) [44]	>65	156	84		28	44 (surgical)		3 years
Mayer et al. (2011) [45]	>60	18				18		6 mo
Hénaux et al. (2011) [46]	>80	11				11		2 mo
Ardeshiri et al. (2013) [11]	>70	28				18	10	24 mo
Chapman et al. (2013) [12] Fehlings et al. (2013) [47] Smith et al. (2013) [48] Vaccaro et al. (2013) [49]	>65	322	157 (nonsurgical)			165 (surgical)		24 mo
Kohlhof et al. (2013) [13]	>62	24				24		6 weeks
Molinari et al. (2013) [14]	>65	26					26	3 mo
Steltzlen et al. (2013) [15]	>65	9				7	2	24 mo

**Table 2:** Means for survival time of all included cases with regard to treatment allocation.

Treatment	*N*	Mean survival ± SE	95% CI
Nonsurgical	533	67 ± 3 months	62–72
Surgical	751	87 ± 4 months	80–95
Overall	1284	83 ± 3 months	78–88

**Table 3:** Nonunion rate for 640 included cases with regard to treatment.

Treatment	*N*	Nonunion	Proportion
Collar	154	60	39%
Halo	73	30	41%
Anterior	293	79	27%
Posterior	149	17	11%
